# Using fecal immmunochemical cartridges for gut microbiome analysis within a colorectal cancer screening program

**DOI:** 10.1080/19490976.2023.2176119

**Published:** 2023-02-16

**Authors:** Stefanie Brezina, Martin Borkovec, Andreas Baierl, Fabienne Bastian, Andreas Futschik, Nikolaus Gasche, Thomas Gruenberger, Michael Hallas, Christian Jannsen, Gernot Leeb, Rebecca Lutz, Barbara Sladek, Andrea Gsur

**Affiliations:** a Center for Cancer Research, Medical University of Vienna, Vienna, Austria; b Department of Statistics and Operations Research, University of Vienna, Vienna, Austria; c Department of Surgery, Clinic Favoriten, Vienna, Austria; d Department of Applied Statistics, Johannes Kepler University Linz, Linz, Austria; e Biome Diagnostics GmbH, Langenzersdorf, Austria; f Institute of Pathology and Bacteriology, Clinic Favoriten, Vienna, Austria; g Department of Internal Medicine, Hospital Oberpullendorf, Oberpullendorf, Austria

**Keywords:** Microbiome, colorectal cancer, 16S rRNA sequencing, FIT, NORGEN

## Abstract

The colorectal cancer (CRC) screening program B-PREDICT is an invited two-stage screening project using a fecal immunochemical test (FIT) for initial screening followed by a colonoscopy for those with a positive FIT. Since the gut microbiome likely plays a role in the etiology of CRC, microbiome-based biomarkers in combination with FIT could be a promising tool for optimizing CRC screening. Therefore, we evaluated the usability of FIT cartridges for microbiome analysis and compared it to Stool Collection and Preservation Tubes. Corresponding FIT cartridges as well as Stool Collection and Preservation Tubes were collected from participants of the B-PREDICT screening program to perform 16S rRNA gene sequencing. We calculated intraclass correlation coefficients (ICCs) based on center log ratio transformed abundances and used ALDEx2 to test for significantly differential abundant taxa between the two sample types. Additionally, FIT and Stool Collection and Preservation Tube triplicate samples were obtained from volunteers to estimate variance components of microbial abundances. FIT and Preservation Tube samples produce highly similar microbiome profiles which cluster according to subject. Significant differences between the two sample types can be found for abundances of some bacterial taxa (e.g. 33 genera) but are minor compared to the differences between the subjects. Analysis of triplicate samples revealed slightly worse repeatability of results for FIT than for Preservation Tube samples. Our findings indicate that FIT cartridges are appropriate for gut microbiome analysis nested within CRC screening programs.

## Introduction

Colorectal cancer (CRC) is the third leading cancer-related cause of death worldwide and represents a major public health issue.^1^ In Austria, the CRC incidence rate is observed in the lower third within the European Union with about 4.500 new cases diagnosed each year.^2^ Moreover, recent data indicate that the incidence of CRC is increasing, especially among younger adults.^3^ Therefore, CRC has become an important and challenging global public health problem, in which the detection of cancer in early stages is of high importance. The natural history of sporadic CRC usually involves slow progression from precancerous polyps to cancer, which offers opportunities for screening and early detection.^4^ Early detection of CRC is an important issue since stage at diagnosis remains the most important prognostic factor.^5^ As CRC is one of the most preventable cancers, population-wide screening programs are recommended in many countries. Screening programs have the potential to detect early precancerous lesions and perform endoscopic removal of adenomas, thereby contributing to the reduction of CRC incidence and mortality.^6–8^

In the ongoing “Colorectal Cancer Study of Austria” (CORSA) participants are recruited in cooperation with the province‐wide screening project “Burgenland Prevention Trial of Colorectal Disease with Immunological Testing” (B‐PREDICT), since 2003.^9^ B-PREDICT, conducted in the Austrian federal state Burgenland, is an invited two-stage screening project for individuals aged between 40 and 80 using a fecal immunochemical test (FIT) for initial screening. Participants with a positive test are offered a diagnostic colonoscopy. During their clinical appointment, these participants are asked to take part in CORSA, sign a written informed consent, complete questionnaires and provide an EDTA blood sample and a stool sample for the CORSA biobank.

The economic burden of CRC in Austria was estimated €157 million per year. These costs account only for general healthcare costs as well as nursing expenses. Informal costs, costs of unpaid patient care provided by friends and relatives, lost earnings due to illness and premature death are not included, but are known to account for a major proportion of CRC-related costs (about 60%).^10^ These figures highlight that non-healthcare costs contribute even more to the socioeconomic burden of CRC and that healthcare costs, cost-effectiveness, and success of cancer treatment are interrelated.

Nowadays, the preferred approach in testing for occult blood in feces used for CRC screening programs is the FIT, despite its relatively low specificity and sensitivity. Commonly used FIT test have shown low sensitivity for precancerous lesions (12.3–32.4%)^11^ and early-stage cancer (40%).^12^ Additionally, FIT tests may show false-negative results due to smoking status or advanced age, both of which are well-known risk factors for CRC, causing some cases to be missed. Taken together, there is an urgent demand for novel noninvasive biomarkers **–** in addition to FIT – to identify those individuals who are more likely to benefit from screening colonoscopy and those who need an earlier or more frequent colonoscopy. The combination of conventional screening methods such as FIT with microbiome-based methods could be a promising tool for early detection of CRC. There is some evidence of carcinogenic mechanisms induced by bacteria^13,14^ and therefore it has been hypothesized that the gut microbiome could play an important role in the development and progression of CRC. Specific changes in the microbiome occur during different stages of colorectal neoplasia, from adenomatous adenomas to early-stage cancer, to metastatic disease, supporting an etiologic and diagnostic role for the microbiome.^15,16^

An important issue in microbiome studies is the sample collection methodology. Although, recent studies have demonstrated that gut-based microbial DNA isolated from FIT cartridges can replace naïve stool samples for microbiome analysis, there is little consent in standard fecal sample collection methods.^17–19^ The standardized sample collection methodology, particularly the feasibility of FIT samples for microbiome analyses within CRC screening programs are currently intensively discussed in research networks and consortia focusing on gut microbiome-based biomarkers.

Therefore, we evaluated the microbial reliability, inter- as well as intra-variability and usability of stool samples collected in FIT cartridges and Stool Collection and Preservation Tubes from participants of the screening program B-PREDICT as well as additional volunteer samples.

## Methods

### Questionnaires

CORSA participants and volunteers provided a basic CORSA questionnaire assessing data on body mass index (BMI), smoking history, alcohol consumption, education level, family status, profession, basic dietary habits, information on use of antibiotics and diabetes.

### Fecal sample collection

Participants were instructed to collect stool samples at most three days prior to bowel cleanse and colonoscopy from the same bowel movement and to store them at room temperature until their clinical appointment. In the hospital, all samples were frozen and stored at −80°C until DNA extraction. Two sample collection methods were used: OC-Sensor FIT cartridges (Eiken Chemical Co., Ltd., Tokyo, Japan) and Stool Collection and Preservation Tubes (Norgen Biotek Corp., Ontario, Canada), henceforth referred to as FIT and Norgen, respectively. Each patient provided one FIT as well as one Norgen sample from the same bowl movement.

In addition to participants recruited with the B-PREDICT screening, five volunteers provided FIT cartridges as well as Norgen samples in triplicates. Volunteer samples were collected from the same bowel movement, stored three days on room temperature, and frozen at −80°C until DNA extraction.

### DNA isolation

DNA isolation is performed from FIT cartridge buffers and matching Norgen samples with the beads-based QIAamp PowerFaecal Pro DNA Kit (Qiagen, Hilden, Germany) in combination with a Precellys® 24 homogenizer (VWR International GmbH, Vienna, Austria). 500 µL buffer-stool solution was used as starting material for DNA isolation from each sample. The quality and quantity of the DNA is assessed prior to 16S rRNA sequencing using a NanoDropTM ND-1000 spectrophotometer (VWR International GmbH, Vienna, Austria) and fluorometrically with the QubitTM dsDNA HS Assay Kit (ThermoFisher Scientific, Vienna, Austria).

### 16s rRNA gene sequencing

For the analysis of the bacterial microbiota, the variable V3-V4 region of the eubacterial 16S rDNA gene was amplified. The 16S small subunit ribosomal gene functions as an exclusive highly conserved housekeeping gene, which can be used to determine microbial communities within samples. Sample library preparation was performed according to the Illumina protocol (Illumina, San Diego, USA) followed by sequence analysis on the Illumina MiSeq platform. The gene‐specific sequences used in the given protocol are selected from Klindworth *et al*.^20^ as the most promising bacterial primer pair. Illumina adapter overhang nucleotide sequences are added to the gene‐specific sequences. The full length primer sequences, using standard IUPAC nucleotide nomenclature, to follow the protocol targeting this region are:

16S Amplicon PCR Forward Primer = 5’

TCGTCGGCAGCGTCAGATGTGTATAAGAGACAGCCTACGGGNGGCWGCAG

16S Amplicon PCR Reverse Primer = 5’

GTCTCGTGGGCTCGGAGATGTGTATAAGAGACAGGACTACHVGGGTATCTAATCC

For sequencing the MiSeq Reagent Kit v3 (Illumina, San Diego, USA) enabling a read length up to 2 × 300 bp was utilized.

### Read pre-processing and taxonomic classification

Primers were trimmed and spacer sequences removed from the raw sequencing reads using cutadapt version 3.4. All trimmed reads were brought to a consistent length using cutadapt, with reads shorter than 280bp/278bp (forward/reverse) being discarded.^21^ The pre-processed reads where then analyzed using FIGARO version 1.1.1^22^ with default options to predict the most optimal quality trimming parameters for DADA2 version 1.18.0.^23^ Using the determined cutoff values of 275/169 basepairs and maximum expected errors of 2/1 (forward/reverse), the reads were quality filtered, trimmed to a uniform length, denoised, and merged into amplicon sequencing variants (ASV). Using DECIPHER version 2.18.1 and IdTaxa^24^ as well as dada2, the ASVs were taxonomically classified using the SILVA v138^25^ database. For the taxonomic classification at the species level, only exact matches were used.

### Statistical analysis

We performed our analyses based on ASVs, representing the highest possible resolution, as well as on various taxonomic ranks, representing different levels of aggregation. ASVs present in less than 5% of the analyzed samples were excluded. Microbial abundances were transformed using the center log ratio (CLR) transformation due to the compositional nature of microbiome datasets.^26,27^ The resulting values are scale invariant and therefore count normalization is unnecessary. Since this transformation cannot be calculated for count matrices containing 0-values, all 0s were imputed using the R package zCompositions applying multiplicative simple replacement.^28^

Differences in sample characteristics between FIT and Norgen samples were visualized using violin plots, i.e. density plots displayed vertically like boxplots.^29^ Intra-class coefficients (ICCs)^30^ were calculated for all ASV abundances between FIT and Norgen samples of the patient cohort (ICC^1,3^) and of the volunteers (ICC(3,k)). Consistency was chosen as the relationship considered to be important, since absolute deviations would not decrease the usability of FIT-originated data for risk prediction. However, ICCs for absolute agreement (ICC^1,2^) were calculated utilizing the triplicate samples (FIT as well as Norgen tubes) available from volunteers. This was done for a range of alpha and beta diversities (i.e. by calculating the respective diversity measure and comparing the first components of the Principal Coordinates Analysis (PCoA)) as well as the ASV CLR abundances. Additionally, all the abundance-based ICCs were calculated for each taxonomic rank (species – phylum) in the same way as for the ASVs.

Calculating the Euclidean distance between two samples using the CLR values results in the Aitchison distance, which was used to perform a hierarchical clustering of all samples with Ward’s clustering criterion.^31^ ALDEx2 was used to identify significantly differently abundant ASVs and taxa between FIT and Norgen samples.^32^ P-values were corrected for multiple testing using the Benjamini–Hochberg method considering all tests performed at that specific taxonomic rank as the total number of hypotheses. Effect sizes calculated by ALDEx2 were converted to standardized effect sizes (Cohen’s d).^33,34^

The volunteer samples, which consist of triplicates of each sample type, were used to calculate a linear model to identify the proportions of the sum of squares explained by the subject and the sample type for each ASV identified in at least three samples. The results are presented together with the sum of squared errors in a ternary plot.^35^ Additionally, separate linear models were fitted to samples of each type to identify the sample-type-specific proportion of variance explained by the subject for each ASV present in at least two samples.

All analyses were performed with the statistical programming language R, version 4.1.1^36^ and the R packages ggplot2,^37^ ggpubr,^38^ and ggraph^39^ were used for visualizations. To produce the PCA the base R-package “stats” was used. Results with a p-value smaller 0.05 were deemed statistically significant.

**Figure 1.**
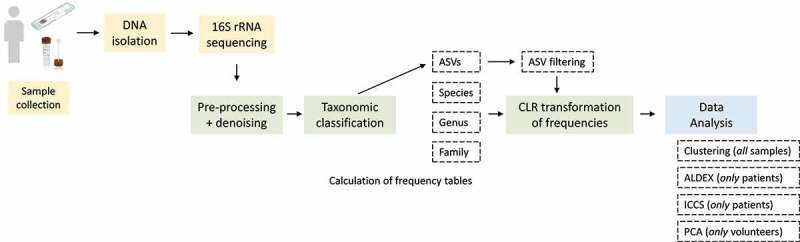
Schematic workflow of the presented study.

Figure 1 is giving a schematic graphical flow chart of the experimental and analytical workflow of the presented study.

### Ethical aspects

Written consent was obtained from all study participants, and all studies were approved by the corresponding Institutional Review Board. Compliance with the 1964 Declaration of Helsinki, the Austrian Drug Law (Arzneimittelgesetz, AMG) and the requirements of Good Clinical Practice of the European Community (CPMP/ICH/135/95) will be ensured. The CORSA study was approved by the institutional review boards (EK 33/2010 and EK 1160/2016).

## Results

### Study participants

Eighty-one participants recruited within B-PREDICT provided a FIT tube and a stool nucleic acid collection and preservation tube (Norgen). The median age of patients was 63.4 years and the median BMI was 27.6. Additionally, five volunteers were recruited with a median age of 30.8 years and a median BMI of 23.1 (Table 1).

**Table 1.** Characteristics of study participants.

	Patients, n = 81	Volunteers, n = 5
**Sex**		
Male, n (%)	49 (60.5)	2 (40.0)
Female, n (%)	32 (39.5)	3 (60.0)
**Age**		
Median (IQR)	63.4 (57.9–70.8)	30.8 (30.1–34.3)
**BMI**		
Median (IQR)	27.6 (24.6–32.0)	23.1 (23.0–23.7)
Underweight, n (%)	1 (1.2)	0 (0.0)
Normal weight, n (%)	22 (27.2)	4 (80.0)
Overweight, n (%)	27 (33.3)	1 (20.0)
Obese, n (%)	31 (38.3)	0 (0.0)
**Smoking habits**		
Smoker, n (%)	15 (18.5)	1 (20.0)
Former smoker, n (%)	34 (42.0)	0 (0.0)
nonsmoker, n (%)	32 (39.5)	4 (80.0)
**Alcohol consumption**		
Consumer, n (%)	49 (60.5)	3 (60.0)
Former consumer, n (%)	4 (4.9)	0 (0.0)
nondrinker, n (%)	20 (24.7)	2 (40.0)
(Missing), n (%)	8 (9.9)	0 (0.0)
**Education**		
Elementary school, n (%)	16 (19.8)	0 (0.0)
Secondary school, n (%)	51 (63.0)	0 (0.0)
Higher school, n (%)	9 (11.1)	1 (20.0)
University, n(%)	4 (4.9)	4 (80.0)
(Missing), n (%)	1 (1.2)	0 (0.0)

### Norgen and FIT samples produce similar numbers of reads and sequences

**Figure 2.**
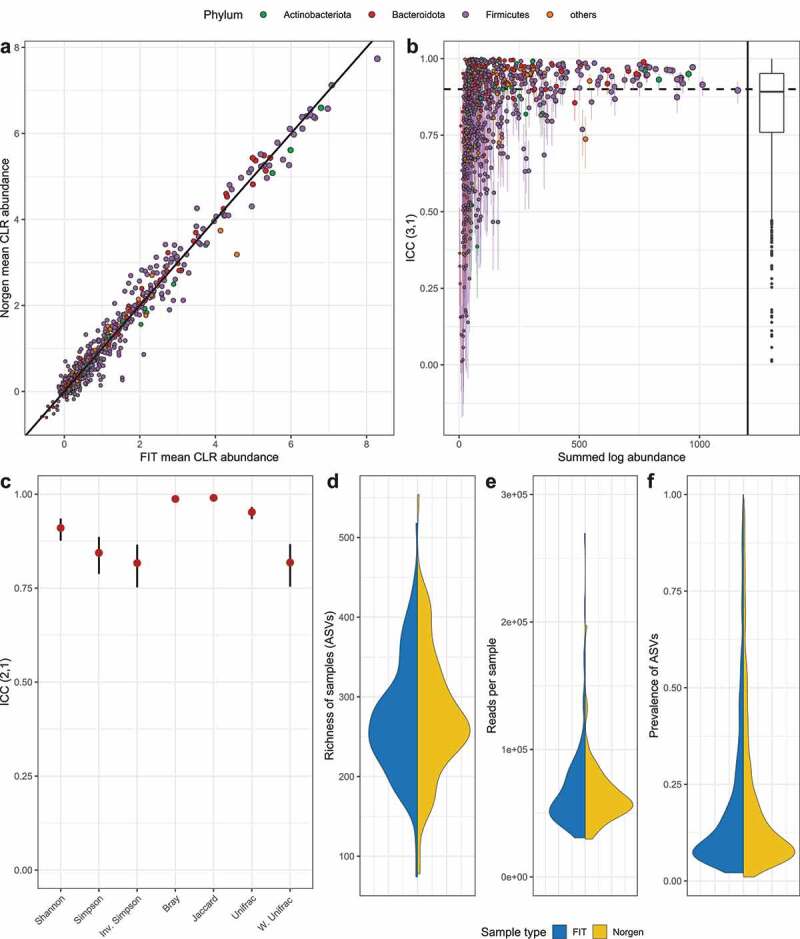
Figure based on patient samples. ASVs are colored according to their phylum and sized according to their abundance. A: Scatterplot of ASVs with mean center log ratio (CLR) transformed abundances of FIT and Norgen samples B: Scatterplot showing the relationship between each ASV‘s ICC estimate (with confidence interval) and the sum of the logarithms of its abundances. ICCs above 0.9 (dashed line) indicate excellent reliability. A boxplot of the ICCs is provided additionally. C: ICC estimates and confidence intervals for all calculated metrics computed on FIT and Norgen samples. Table 2 is giving all calculated beta diversities. The ICC of the Shannon, Simpson and Inverse Simpson index are all above 0.75, with the Shannon index providing the highest reliability between FIT and Norgen. The Bray-Curtis dissimilarity and the Jaccard index results are almost in perfect agreement. Unweighted UniFrac also displays an excellent ICC, while the weighted version results in only good reliability. D, E, F: Violin plots comparing Norgen and FIT samples based on the richness of the samples (d), number of reads per sample (e) and the prevalence of ASVs identified in at least 5% of the samples (f).

The denoised reads contained 6,097 ASVs. Taxonomic classification of all ASVs yielded 241 species, 240 genera, 80 families, 47 orders, 21 classes, and 14 phyla, with varying proportions of reads classified at each taxonomic rank (Fig. S1). The median richness was 263 ASVs for FIT samples and 265 ASVs for Norgen samples (Figure 2d) and the median number of reads after filtering was 60,283 for FIT and 59,266 for Norgen (Figure 2e).

**Table 2.** Beta diversities figures.

ICC	Lower bound	Upper bound	Measure
0.910	0.876	0.935	Shannon
0.817	0.752	0.866	Inv. Simpson
0.844	0.788	0.866	Simpson
0.987	0.982	0.911	Brav
0.990	0.986	0.993	Jaccard
0.952	0.934	0.966	Unifrac
0.818	0.754	0.867	W. Unifrac

Of the identified ASVs 1,029 (16.9%) were detected in more than 5% of the samples after filtering. The median prevalence of these ASVs (i.e. percentage of samples in which an ASV was detected) was 11.5% in FIT samples and 12.5% in Norgen samples (figure 2f).

### Average CLR abundances similar between FIT and Norgen

The average CLR abundances of ASVs display high similarity between the FIT and the Norgen samples (Figure 2a). Among the 10 ASVs with the highest differences between sample types, seven are more abundant in FIT samples. Of these, the highest differences can be observed for an ASV belonging to the genus Escherichia-Shigella of the Phylum Proteobacteria and the rest belong to the genera Enterococcus, Lactococcus, Streptococcus, Leuconostoc. Of the three ASVs with higher abundance in Norgen samples one belongs to the genus Oscillibacter and two could not be classified on the genus rank. Complete results, including the comparisons on each taxonomic rank are available in Table S1 and Fig. S1.

### ICCs positively associated with abundance of ASVs

The ASV-specific ICCs between the FIT and Norgen samples of the patients (Figure 2b) display a positive association with the summed log abundances of the ASV. Low summed log abundances are in many cases accompanied by low ICCs and large confidence intervals. This indicates, that the estimates lack in precision for many of the rarer ASVs. Overall, the ICCs’ first quartile is 0.759, the median is 0.892, and the third quartile is 0.951. A common interpretation is, that an ICC higher than 0.75 indicates good reliability and an ICC higher 0.9 indicates excellent reliability.^30^ Fig. S1 provides visualizations of this analysis for taxonomic ranks from species to phylum and Table S2 contains complete ICC estimates and confidence intervals for all bacterial taxa and ASVs. These results confirm an association between abundances and reliability. Additionally, these results indicate higher reliability for higher ranks. This is probably due to the fact that higher ranks result in deeper aggregation and higher proportions of classified reads. ICCs were also estimated based on the volunteer samples, which consisted of triplicates for each sample type. The “between FIT and Norgen” ICCs were therefore calculated based on the means of the respective samples. Additionally, this allowed for the estimation of the ICCs within the FIT and within the Norgen samples (Fig. S2). However, these were calculated as being obtained from three separate random raters (i.e. triplicate samples), resulting in lower and less stable estimates than the “between FIT and Norgen” ICCs, making a direct comparison of these results impossible. Nevertheless, this analysis shows that even separate stool samples of the same sample type and from the same subject contain noteworthy heterogeneity. The ICC estimates for the alpha and beta diversities and their confidence intervals can be seen in Figure 2c. In addition, Table 2 is giving all beta diversities. The Shannon, Simpson, and Inverse Simpson indices all display ICCs above 0.75, with Shannon providing the highest reliability between FIT and Norgen. In the case of the beta diversities, the Bray-Curtis dissimilarity and the Jaccard index result in almost perfect agreement. Unweighted UniFrac also displays an excellent ICC, while the weighted version results in only good reliability.

### Samples form subject-specific clusters

**Figure 3.**
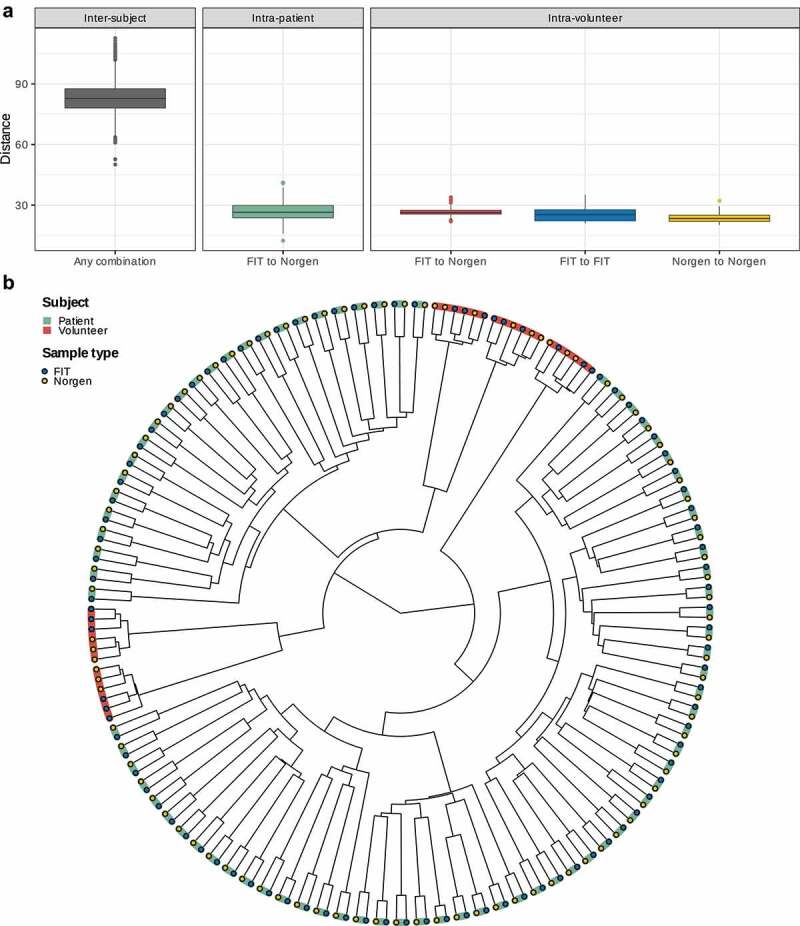
A: [gray box] Boxplot of distances between samples of different subjects (regardless of sample type), [green box] between FIT and Norgen samples of the same patient and [red box] between FIT and Norgen samples, [blue box] between FIT samples and [yellow box] between Norgen samples of the same volunteer. B: Result of a hierarchical clustering algorithm based on the distances between all samples. Samples originating from the same subject are connected with colored bars.

The inter-subject distances (i.e. all possible distances between two samples from different subjects) displayed a median of 82.4, a maximum of 113.0 and a minimum of 50.1, which is higher than the maximum of all intra-subject distances, namely 41.2. The intra-subject distances consist of the distances between the FIT and the Norgen samples of each patient (1 distance per patient; median = 26.5) and each volunteer (9 distances per volunteer; median = 26.4) as well as the distances between the FIT (3 distances per volunteer; median = 25.4), respectively, Norgen (3 distances per volunteer; median = 23.5) triplicates of each volunteer (Figure 3a). The intra-volunteer distances were significantly different (Kruskal-Wallis test: p = < 0.001) and of the subsequent pairwise tests only the comparison between “FIT to Norgen” distances and “Norgen to Norgen“ distances reached statistical significance (Wilcoxon test: p = < 0.001). Based on these distances, a hierarchical clustering was performed on all samples. All samples clustered together according to the subject who provided them before being joined with samples of other subjects (Figure 3b).

### PCA of volunteer samples reveals no separability of FIT and Norgen samples

**Figure 4.**
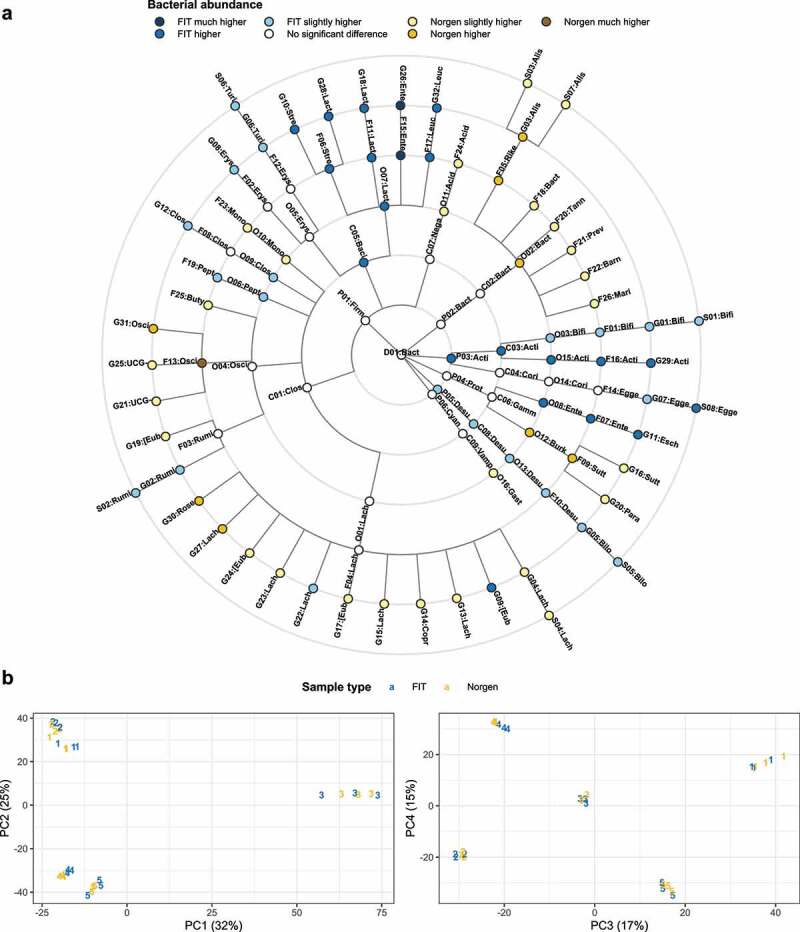
A: Taxonomic tree displaying significant differences between FIT and Norgen samples based on the ALDEX analysis. Taxa are labeled with an ID and the first letters of their name. Full taxa names are given in Table 3. B: Scatterplots of the first four principal components extracted from the volunteer samples. Each of the five volunteers is represented by a number.

The first four principal components of the ASVs CLR abundances in the volunteer samples are shown in Figure 4b and reveal no sample type-specific clusters. Only the samples recruited from volunteer no. 4 display some slight separability between FIT and Norgen samples. However, all other samples cluster randomly around a subject-specific center, regardless of the sample type.

**Table 3.** List of full names for taxa displayed in Figure 3a. Effect sizes were calculated with ALDEx2 and are only shown for significant (after p-value correction) differences. Negative values indicate higher abundances in FIT samples, while positive values correspond to higher abundances in Norgen samples.

Label	Name	Effect	Label	Name	Effect
*Domain*			F19:Pept	Preptostreptococcaceae	−0.09
D01:Bact	Bacteria		F20:Tann	Tannerellaceae	0.08
*Phylum*			F21:Prev	Prevotellaceae	0.10
P01:Firm	Firmicutes		F22:Barn	Barnesiellaceae	0.07
P02:Bact	Bacteroidota		F23:Mono	Monoglobaceae	0.07
P03:Acti	Actinobacteriota	−0.18	F24:Acid	Acidaminocococcaceae	0.08
P04:Prot	Proteobacteria		F25:Buty	Butyricicoccaceae	0.11
P05:Desu	Desulfobacterota	−0.14	F26:Mari	Marinifilaceae	0.11
*Class*			*Genus*		
C01:Clos	Clostridia		G01:Bifi	Bifidobacterium	−0.15
C02:Bact	Bacteroidia		G02:Rumi	Ruminococcus	−0.12
C03:Acti	Actinobacteria	−0.17	G03:Alis	Alistipes	0.20
C04:Cori	Coriobacteria		G04:Lach	Lachnospira	0.15
C05:Baci	Bacilli	−0.25	G05:Bilo	Bilophila	−0.09
C06:Gamm	Gammaproteobacteria		G06:Turi	Turicibacter	−0.11
C07:Nega	Negativicutes		G07:Egge	Eggerthella	−0.11
C08:Desu	Desulfovibrionia	−0.13	G08:Erys	Erysipelotrichaceae UCG-003	−0.09
*Order*			G09:[Eub	[Eubacterium] hallii group	−0.14
O01:Lach	Lachnospirales		G10:Stre	Streptococcus	−0.27
O02:Bact	Bacteroidales	0.18	G11:Esch	Escherichia-Shigella	−0.27
O03:Bifi	Bifidobacteriales	−0.11	G12:Clos	Clostridium sensu stricto 1	−0.06
O04:Osci	Oscillospirales		G13:Lach	Lachnoclostridium	0.07
O05:Erys	Erysipelotrichales		G14:Copr	Coprococcus	0.06
O06:Pept	Pepostreptococcales-Tissierellales	−0.10	G15:Lach	Lachnospiraceae NC2004 group	0.05
O07:Lact	Lactobacillales	−0.34	G16:Sutt	Sutterella	0.14
O08:Ente	Enterobacterales	−0.26	G17:[Eub	[Eubacterium] eligens group	0.12
O09:Clos	Clostridiales	−0.05	G18:Lact	Lactobacillus	−0.29
O10:Mono	Monoglobales	0.06	G19:[Eub	[Eubacterium] siraeum group	0.12
O11:Acid	Acidaminococcales		G20:Para	Parasutterella	0.09
O12:Burk	Burkholderiales	0.28	G21:UCG-G-	UCG-005	0.09
O13:Desu	Desufovibrionales	−0.08	G22:Lach	Lachnospiraceae FCS020 group	−0.15
O14:Cori	Coriobacteriales		G23:Lach	Lachnospiraceae UCG-004	0.11
O15:Acti	Actinomycetales	−0.20	G24:[Eub	[Eubacterium] xylanophilium group	0.13
*Family*			G25:UCG-	UCG-005	0.15
F01:Bifi	Bifidobacteriaceae	−0.11	G26:Ente	Enterococcus	−0.52
F02:Erys	Erysipelatocotridiacea		G27:Lach	Lachnospiraceae UCG-001	0.20
F03:Rumi	Ruminococcaceae		G28:Lact	Lactococcus	−0.33
F04:Lach	Lachnospiraceae		G29:Acti	Actinomyces	−0.23
F05:Rike	Rikenellaceae	0.25	G30:Rose	Roseburia	0.20
F06:Stre	Streptococcaceae	−0.21	G31:Osci	Oscillibacter	0.21
F07:Ente	Enterobacteriaceae	−0.26	G32:Hold	Holdemania	0.11
F08:Clos	Clostridiaceae		G33:Leuc	Leuconostoc	−0.27
F09:Sutt	Sutterellaceae	0.33	*Species*		
F10:Desu	Desulfovibrionaceae	−0.08	S01:Bifi	Bifidobacterium longum	−0.09
F11:Lact	Lactobacillaceae	−0.27	S02:Rumi	Ruminococcus bromii	−0.10
F12:Erys	Erysipelotrichaceae		S03:Alis	Alistipes putredinis	0.10
F13:Osci	Oscilloscpiraceae	0.43	S04:Lach	Lachnospira pectinoschiza	0.14
F14:Egge	Eggerthellaceae		S05:Bilo	Bilophila wadsworthia	−0.08
F15:Ente	Enterococcaceae	−0.49	S06:Turi	Turicibacter sanguinis	−0.12
F16:Acti	Actinomycetaceae	−0.19	S07:Alis	Alistipes obesi	0.10
F17:Leuc	Leuconostocaceae	−0.21	S08:Egge	Eggerthella lenta	−0.13
F18:Bact	Bacteroidaceae	0.13			

### Differential abundance detected at various taxonomic ranks

Bacterial abundances of the patients’ FIT and Norgen samples were compared at all taxonomic ranks (species to phylum) and the significant results are presented in Figure 4a as a taxonomic tree. Some branches of the tree display consistent differences between the sample types. For example, all significantly differentially abundant taxa belonging to the phylum *Actinobacteriota* or the class *Bacilli* are more abundant in FIT samples, while all the significant taxa belonging to the class *Bacteroidales* are more abundant in the Norgen samples. However, there are also inconsistent branches, like the family of *Lachnospiraceae*, which contain both, genera more abundant in FIT and genera more abundant in Norgen samples. Complete results of the ALDEx2 analysis are available in Table S3.

### Sample type explains only small proportion of sum of squares

**Figure 5.**
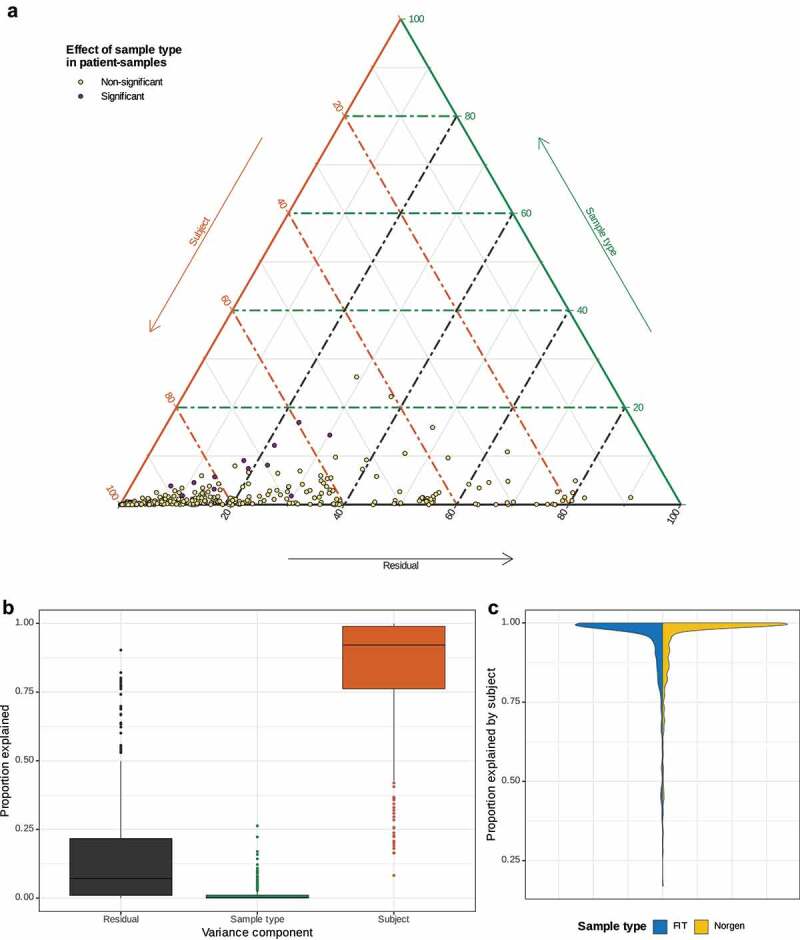
Analyses of triplicate samples of the five volunteers (3x FIT, 3x Norgen). A: Ternary plot displaying the variance components of each ASV as a single point based on a linear model with subject and sample type as explanatory variables. Considering only ASVs detected in at least three volunteer samples. ASVs are colored according to the results of the corresponding significance tests on the patient samples. B: Boxplots of the variance components used in A. C: Violin plots comparing the proportions explained by subject in separate models for FIT and Norgen. Only ASVs detected in at least three samples of the respective sample type were used.

Linear models were fitted on the CLR abundances of the volunteer samples for all ASVs detected in at least 3 of the 30 samples. The resulting proportions of sum of squares explained by subject and sample type as well as the residual proportions are shown in a ternary plot in Figure 5a and the corresponding boxplots in Figure 5b. This shows that most of the variance in the ASVs’ CLR abundances can be explained by the subject compared to only small amounts which are explained by the sample type. Some ASVs display a high proportion of residual variance, which overall constitutes a much bigger issue for the repeatability of results. This is also evident from the results of separate models for FIT and Norgen using ASVs detected in at least three samples of the respective type (Figure 5c). This model specification shows that the amounts of variance explained by subject are slightly lower (i.e. residual variance is higher) for FIT than for Norgen. For both sample types, there is a peak at proportions near 1, which is slightly less pronounced for FIT and corresponds to a lower mean of 0.930 for FIT, compared to 0.936 for Norgen.

## Discussion

Several CRC screening programs such as B-PREDICT implemented a two-stage screening, using FIT for the initial screening. The combination of conventional screening methods such as FIT with microbiome-based methods could be a promising tool for optimizing early detection of CRC. To investigate the usability of FIT samples for gut microbiome analysis, we compared FIT samples as well as stool samples collected in conventional Preservation Tubes (Norgen) from participants of the CRC screening program B-PREDICT and additional volunteers.

Our findings are mostly in accordance with previously published studies. Multiple prior studies concluded that microbial composition and diversity were largely explained by between-participants differences and only marginally by the collection methods.^40,41^ Furthermore, different studies demonstrated that FIT tubes used for fecal occult blood sample collection have the potential to be used for sample collection for microbiome studies.^42,43^ Besides others,^44,45^ Gudra *et al*. have evaluated fecal sample stability in the commonly used OC-Sensor (Eiken Chemical, Tokyo, Japan), the same FIT tube applied in the B-PREDICT study, under various storage conditions employing two different sequencing platforms. They did not find a significant difference between immediately frozen samples and samples stored for 2 days at 4°C and for 2 days at 20°C.^46^ Masi and colleagues expanded upon these finding by investigating the performance of FIT samples in the English Bowel Cancer Screening Programme to understand the role of gut microbiome in colorectal neoplasia holds great promise. In concordance with other studies^16,47^ exploring the potential of FITs for microbiome sequencing, they concluded that fecal microbiome diversity and taxonomic profiles were consistent across test conditions.^48^

Sinha *et al*. demonstrated in their study comprising 20 volunteers that the Fecal Occult Blood Test (FOBT) is a reasonable sample collection method with optimal stability and reproducibility for 16S rRNA microbiome profiling.^17^ Furthermore, a recent study by Zouiouich and colleagues, investigating the impact of sample collection and storage method on the accuracy and stability of 16S rRNA sequencing, could show that stability ICCs were high for FIT tubes that were collected in course of a colorectal cancer screening setting. The authors concluded that commonly stool collection cards and different types of FIT tubes are acceptable tools for microbiome measurements and have the utility for developing microbiome-focused cohorts nested within screening programs.^19^ In addition, a further study comparing microbiome stability and accuracy across different fecal sample collection methods, commonly used in ongoing CRC screening program, concluded that the interindividual variability was much higher than the variability introduced by the collection method. However, they authors found that different types of FIT tubes did not seem to perform equally in terms of relative abundance of phyla and genera, which support observations from previous studies.^16^ Furthermore, a recent study using FIT as well as fresh frozen facal samples of 30 volunteers of an Estonian screening program concluded that the variation between individuals was greater than the differences introduced by the collection strategy and that the vast majority of the genera were stable for up to 7 days.^49^ Moreover, a study by Grobbee and colleagues could show that fecal microbial content can be measured in FIT samples and remains stable for over six days. Results of their qPCR measurements of positive FIT samples illustrated that the total bacterial load was higher in colorectal cancer patients and patients diagnosed with a high-grade dysplasia.^50^

Our results indicate, that the microbial communities obtained from Norgen samples and FIT tubes are highly similar, mainly differing in two specific attributes. Norgen samples display a lower residual variance, i.e. higher repeatability. We have shown, that the median FIT to FIT distance is 8.0% higher than the median Norgen to Norgen distance, representing the increase in unaccounted variation across the complete microbiome profile. Furthermore, there are differences in abundances of several taxa due to sample type. Although the overall effect of the sample type on the microbiome profile is only slight, significant differences between FIT and Norgen were detected for some taxa within B-PREDICT participants. These results are supported by the analysis of triplicate volunteer samples. However, it is also evident that even for the ASVs affected by the sample type, the resulting microbial abundance is much more strongly influenced by the subject. Subject-specific agreement of ASV-abundances is only slightly affected by sample type and clearly more negatively affected by residual variance, which probably arises due to issues like zero-inflation^51^ and false-positive detection, which impact low-abundance taxa more strongly and are inherent to microbiome analysis. Generally, taxa with low abundances are associated with lower agreement and lower ICCs. Therefore, increasing the taxonomic rank on which an analysis is performed (i.e. from genus to family) leads to results indicating higher reliability. In contrast to the majority of already published data we could prove that FIT samples, a broadly used pre-screening test in CRC screening programs, hold the potential to be applied as additional diagnostic strategy to detect shifts in microbiome profiles and thereby may guide individual patient surveillance. Overall, we could show that FIT samples can be used for profiling the microbiota in a CRC screening setting.

A limitation of our study is that no homogenization of sample material during sampling was performed, thereby inevitably introducing variation into samples from the same subject. To assess a baseline of this variation, triplicate samples were obtained from volunteers and incorporated into the analysis. However, FIT samples analyzed in the present study were obtained in course of the regular B-PREDICT process representing a usual sampling procedure within a CRC screening. A further limitation of the presented study is the application of 16S rRNA sequencing depending on a single gene, the 16S small subunit ribosomal RNA gene, known to be limited by short read lengths obtained as well as the limitation to two different hypervariable regions V3 and V4.^52^ However, as the main objective of the present study was to evaluate the usability of FIT cartridges for microbiome analysis in a colorectal cancer screening setting, we selected 16S rRNA sequencing being proven as a reliable and efficient option for taxonomic classification. Furthermore, 16S rRNA sequencing has enhanced microbiome studies by improving accuracy and making tests cost-effective holding the potential to be applied as a routine diagnostic method to detect shifts in microbiome profiles.^53^

Our findings, taken together with previous studies, demonstrate the potential of FIT, as obtained through a national CRC screening program, to provide a convenient, representative, and cost-effective means of studying fecal microbiota in a large population.

Besides the validation of our results in larger international study cohorts, our next research steps will include an association study aiming to link microbiome profiles to clinical outcomes and patient histories. Due to the medical trend moving toward personalized medicine, there is a huge demand of novel noninvasive biomarkers to stratify patients according to their risk to develop cancer and to tailor individual surveillance. Results from our ongoing work will contribute to the improvement of targeted and cost-effectiveness medicine by combining conventional CRC screening methods such as FIT with innovative microbiome-based methods, and the identification of better biomarkers for patient risk stratification, needed to guide clinical follow-up, surveillance and targeted screening. Furthermore, as sequencing technologies are becoming cheaper, clinics will integrate genetic analysis into their routine. Microbiome analysis is expected to play a main role in optimizing future clinical routine.

## Conclusions

In conclusion, the present study supports previous findings indicating that microbial data obtained from different collection methods are relatively stable and may be an appropriate method to collect fecal samples for gut-based microbiome profiling in CRC screening studies to optimize current CRC screening. However, validation in larger studies as well as association studies, linking microbiome profiles and clinical outcomes, are warranted.

## Supplementary Material

Supplemental Material

## Data Availability

Raw sequencing data and patient metadata are available at the NCBI Sequence Read Archive (BioProject PRJNA801143). R-scripts used in the analysis can be found hear: https://github.com/martin-borkovec/corsa-microbiome.

## Abbreviations

ASV: Amplicon sequence variants; B-PREDICT: Burgenland Prevention Trial of Colorectal Cancer Disease with Immunological Testing; BMI: Body mass index; CRC: Colorectal cancer; CORSA: Colorectal Cancer Study of Austria; CLR: Centre log ratio; FIT: Fecal immunochemical test; FOBT: Fecal occult blood test; ICC: Intraclass correlation coefficient

## Declarations

***Ethical Approval and Consent to participate***

Written consent was obtained from all study participants and all studies were approved by the corresponding Institutional Review Board. Compliance with the 1964 Declaration of Helsinki, the Austrian Drug Law (Arzneimittelgesetz, AMG) and the requirements of Good Clinical Practice of the European Community (CPMP/ICH/135/95) will be ensured. The CORSA study was approved by the institutional review boards (EK 33/2010 and EK 1160/2016).

## Preprint

The present manuscript has been uploaded as Author’s Original Manuscript for preprint at https://doi.org/10.21203/rs.3.rs-1294888/v1.
