# Supplementary material for: Using fecal immmunochemical cartridges for gut microbiome analysis within a colorectal cancer screening program
Source: Gut Microbes. 2023 Feb 16;15(1):2176119. doi: 10.1080/19490976.2023.2176119 (PMC9980522; doi:10.1080/19490976.2023.2176119)
Supplement: Supplemental Material [file KGMI_A_2176119_SM9600.zip › Figures S1_S2_revision2.pdf]

USING FAECAL IMMMUNOCHEMICAL CARTRIDGES FOR GUT MICROBIOME ANALYSIS WITHIN A COLORECTAL CANCER SCREENING PROGRAM

Supplementary Figures

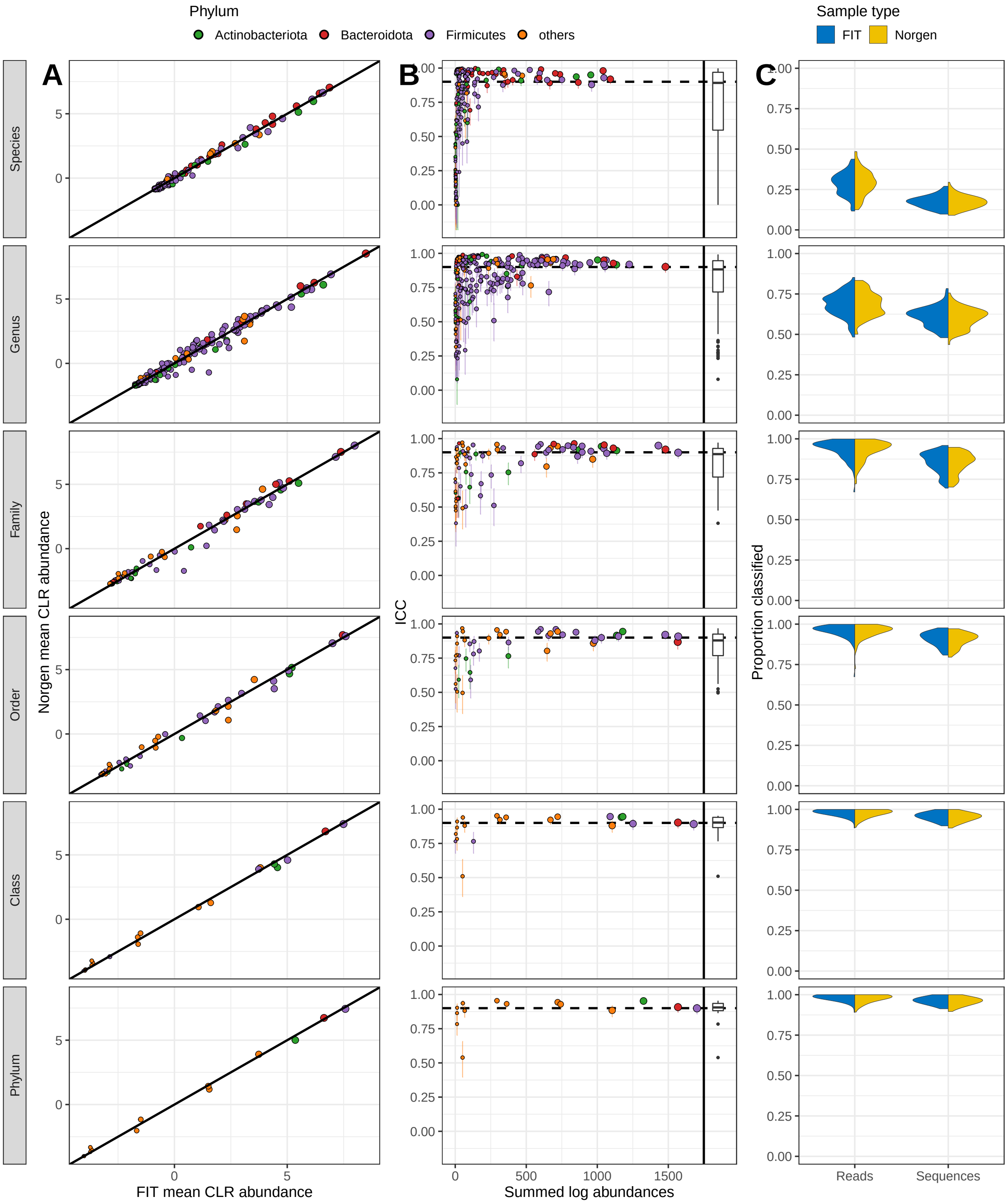

Figure S1: Figure based on patient-samples. ASVs are coloured according to their phylum and sized according to their abundance. A: Scatterplot of ASVs with mean center log ratio transformed abundances of FIT and Norgen samples for each taxonomic rank. B: Scatterplot showing the relationship between each ASV‘s ICC estimate (with confidence interval) and the sum of the logarithms of its abundances for each taxonomic rank. ICCs above 0.9 (dashed line) indicate excellent reliability. Boxplots of the ICCs are provided additionally. C: Violin plots displaying the proportions of reads and sequences classified at each taxonomic rank.

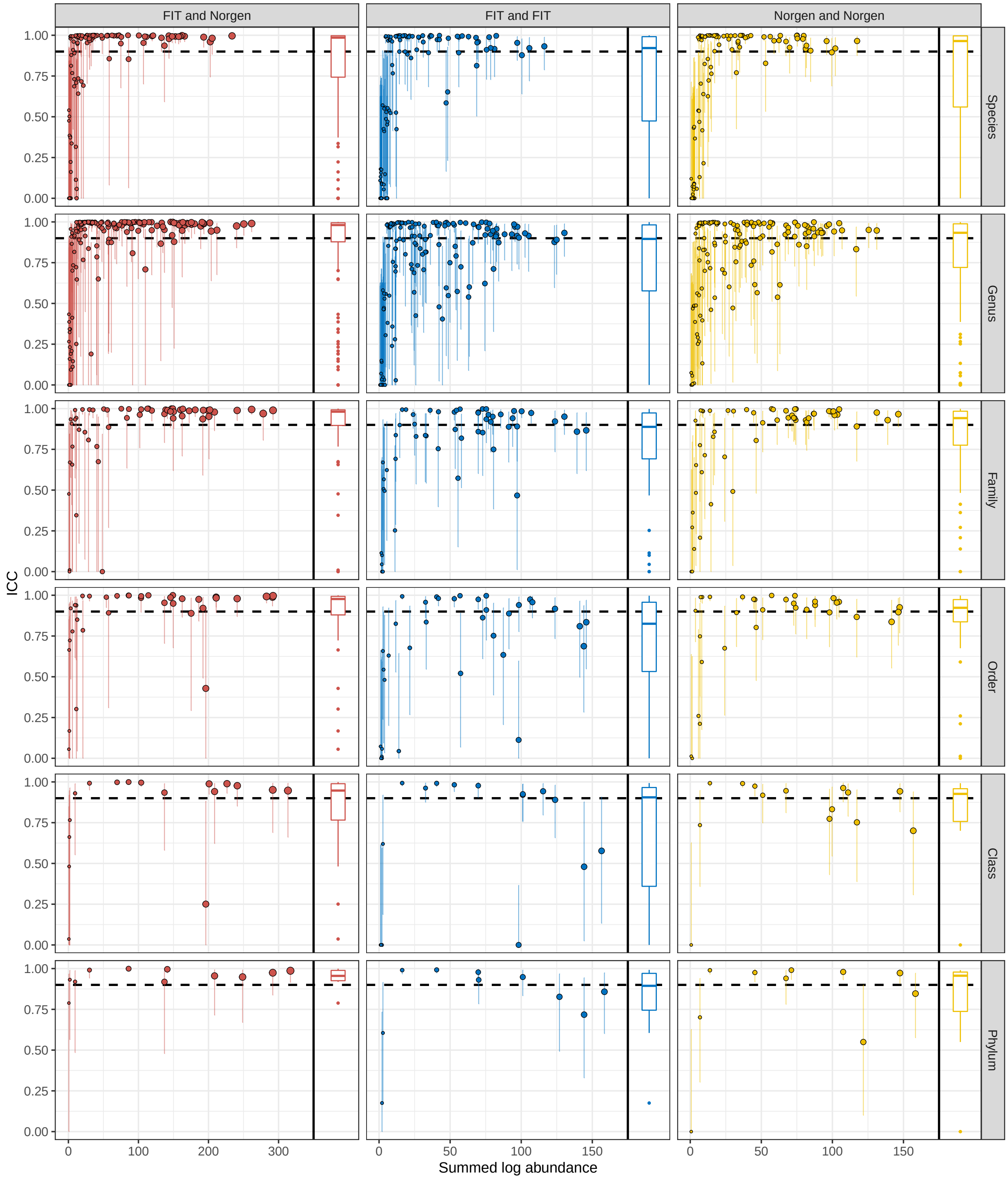

Figure S2: Figure based on volunteer-samples. Scatterplot showing the relationship between each ASV's ICC estimate (with confidence interval) and the sum of the logarithms of its abundances for each taxonomic rank. ICCs above 0.9 (dashed line) indicate excellent reliability. Boxplots of the ICCs are provided additionally. Points are sized according to the ASV's abundance used for the respective analysis.
